# Selective Transarterial Embolization of Post-traumatic Hepatic Artery Pseudoaneurysm and Fistula With Portal Vein

**DOI:** 10.7759/cureus.117552

**Published:** 2026-10-06

**Authors:** Muhammad I Zahoor, Abdulmajeed M Bajunaid, Ahmad J Bosaily, Marei H Alshandeer

**Affiliations:** 1 General Surgery, King Khalid University, Abha, SAU; 2 General Surgery, Saudi Board of General Surgery, Khamees General Hospital, Abha, SAU; 3 General Surgery, American Board of Surgery, King Khalid University, Abha, SAU; 4 General Surgery, Saudi Board of Surgery, King Khalid University, Abha, SAU

**Keywords:** hepatic vascular injury, penetrating trauma, portal vein fistula, pseudoaneurysm, transarterial embolization

## Abstract

Intrahepatic vascular injuries are rare, mostly associated with penetrating trauma, and pose a significant threat to life. Pseudoaneurysm of the hepatic artery and fistula formation with the portal vein is a rare complication that was managed successfully by transarterial embolization in our patient. A victim of gunshot injury presented in a peripheral hospital with injuries to the liver, right kidney, and diaphragm and was managed on principles of damage control surgery. After stabilization, he was transferred to a trauma center where re-exploration was done. Persistent hepatic subcapsular collection led to the diagnosis of hepatic artery pseudoaneurysm and fistula formation with the portal vein on contrast CT scan and was confirmed by angiogram. Transarterial embolization with coils was done, and the collection was drained by a pigtail catheter. The patient recovered fully and showed no complications from embolization. He was discharged in a stable condition.

## Introduction

The liver is commonly injured in blunt and penetrating abdominal trauma. The hemodynamically stable patients are managed conservatively, with success rates of 85% to 94% in blunt hepatic trauma [1]. Intra-hepatic vascular injuries are rare; hepatic pseudoaneurysm occurs in approximately 1.2% of patients [1], mostly associated with penetrating trauma, and poses a significant threat to life demanding urgent diagnosis (CT scan) followed by accurate localization of the bleeding site and prompt management. Gunshot wounds or sharp objects can directly lacerate the artery and can manifest as a transection (partial or complete), occlusion, or a pseudoaneurysm and fistula formation. Intra-hepatic pseudoaneurysm have a weakened, bulging arterial wall and with hepatic parenchyma and hematoma, form a high-pressure blood filled cavity having high risk of rupture. Branches of the hepatic artery are one of the common sites, often presenting days to weeks later. The guidelines of managing major hepatic trauma were continuously updated in the US with frequent use of minimally invasive techniques [2]. Damage control surgery principles may aid management. For both Gunshot wounds and stab wounds, conservative management is adapted for selected patients based on similar general principles [3]. Due to dual blood supply, liver is resistant to ischemia, endovascular embolization in acute hepatic bleeding can provide complete therapeutic success, avoiding the risks and cost of surgery [4]. It is accepted as the standard practice in hemodynamically stable patients with contrast blush on imaging [5].

We report a case of gunshot injury to the abdomen, with intra-hepatic pseudoaneurysm (IHPA) and fistula formation between the posterior segmental branch of the right hepatic artery and the posterior branch of the right portal vein. The patient was treated initially on the principles of damage control surgery at a peripheral hospital, followed by multidisciplinary team management at our trauma center and angioembolization of the IHPA and fistula.

## Case presentation

A 28-year-old man presented to a peripheral hospital with a right-sided thoraco-abdominal gunshot injury with an oblique course from the right lumbar region to the lower chest along the midclavicular line. The patient was conscious but hemodynamically unstable with suspected injury to the liver and intra-abdominal hemorrhage. Following Advanced Trauma Life Support (ATLS) guidelines [6], resuscitation started immediately. Blood samples were taken for routine investigations of hematology, biochemistry, grouping, and cross-matching. The patient was transferred to the operating room, and exploratory laparotomy was performed by the surgical team. Massive hemoperitoneum with grade V injury of the right kidney and large hepatic laceration of segments VI, VII, and VIII extending deeply to the porta hepatis were found. Following principles of Damage Control Surgery, perihepatic packing was done with laparotomy pads into the space between the liver and diaphragm, anterior and lateral abdominal walls, and the hepatic flexure of the colon. Right nephrectomy with repair of the right cupola of the diaphragm was done, and a thoracostomy drain was inserted on the right side for hemopneumothorax. Temporary closure of the abdomen was done, and the patient was transferred to the intensive care unit to continue the resuscitation. In the wake of insecure hemorrhage, no pharmacological prophylaxis for deep venous thrombosis was given, and pneumatic compression continued in the ICU. After two days of aggressive resuscitation, with multiple transfusions, the patient became hemodynamically stable. A pan-CT scan was performed (Figure 1).

**Figure 1:**
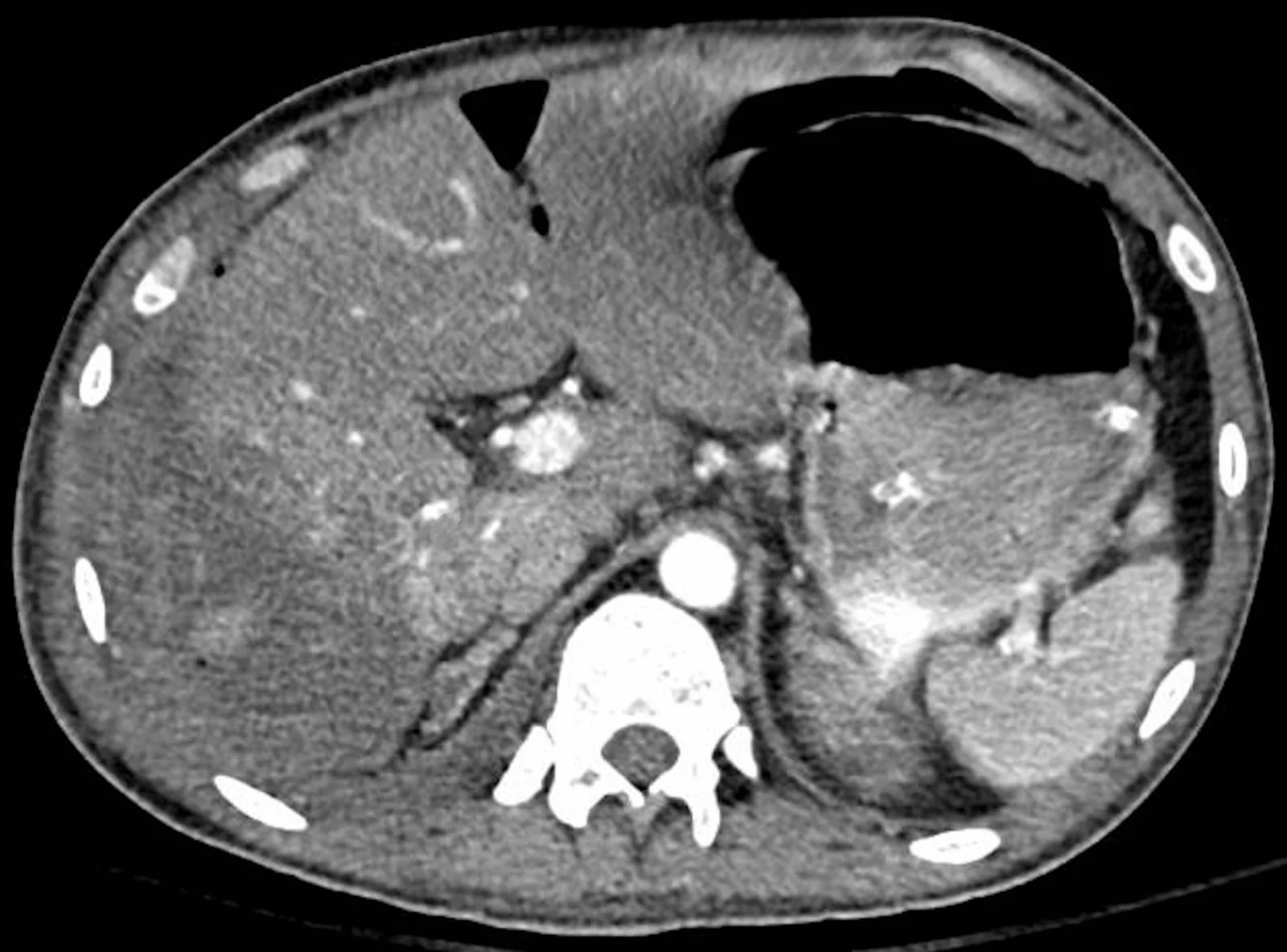
Contrast-enhanced CT liver Deep laceration of the right lobe of the liver, grade IV.

The patient was transferred to the trauma center of the region for the removal of packs and definitive surgery. His abdomen was explored in the operating room for a second look. The trauma team found no active bleeding, and packs were removed. The abdominal cavity was washed with warm saline and closed after placing subhepatic and pelvic drains. Two packed red cells, four units of fresh frozen plasma, and four platelet concentrates were transfused. As CT showed venous thrombosis in the left iliac vein, the team contacted the interventional radiology unit to place an inferior vena cava filter to prevent pulmonary embolism, which was done on the same day (Figure 2).

**Figure 2:**
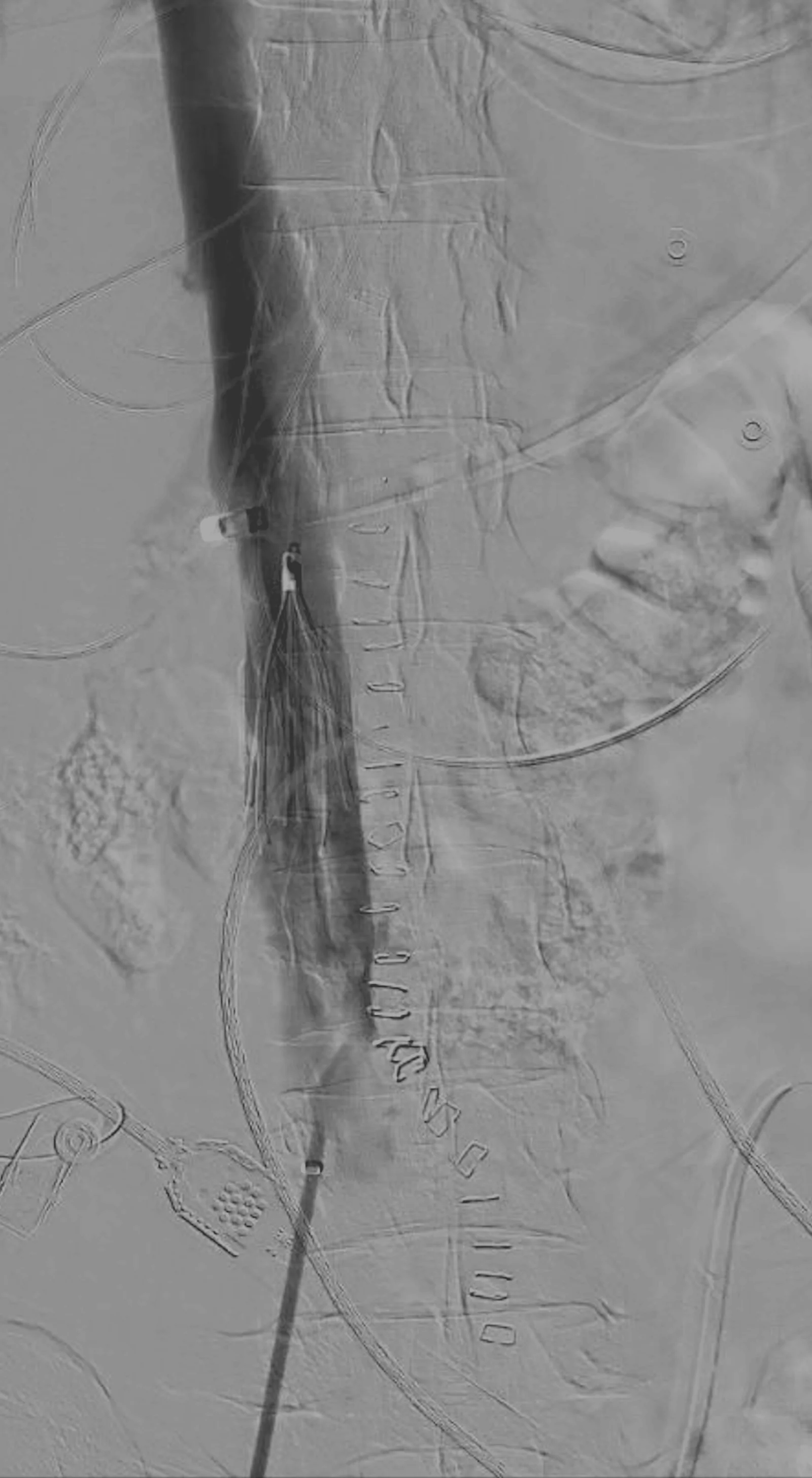
CT angiography and IVC filter placement for partial occlusion of left common iliac vein with venous thrombosis IVC: Inferior Vena Cava

The patient was kept intubated on the mechanical ventilator, nursed, and monitored in the ICU. The follow-up CT scan was done on the 8th postoperative day, which showed a subcapsular hepatic collection (Figure 3).

**Figure 3:**
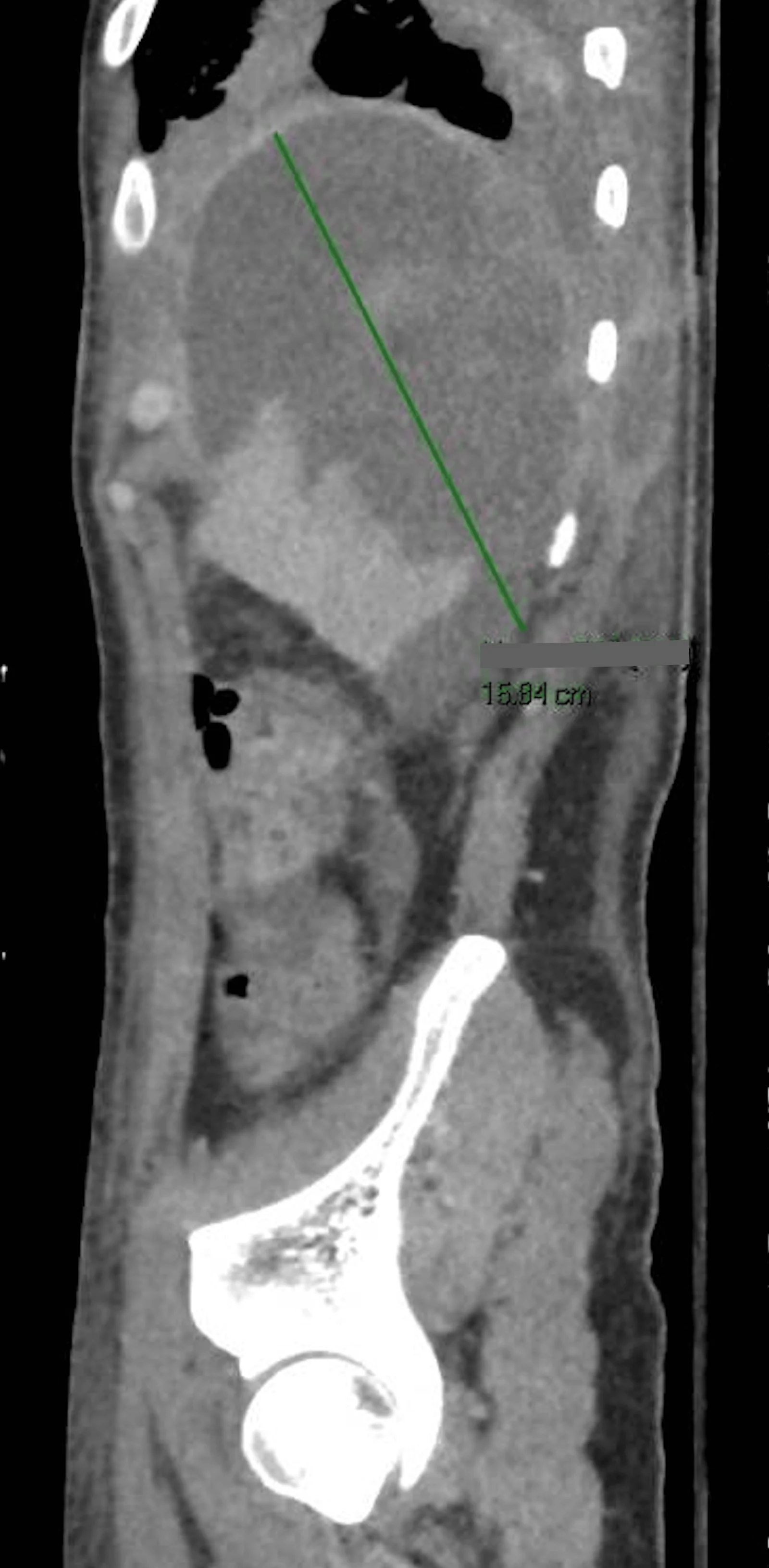
Contrast-enhanced CT liver Subcapsular collection is visible, measuring 10 x 7 x 16 cm in anteroposterior, transverse, and cranio-caudal dimensions, with high-density fluid 24 HU, extending into the subhepatic region inferiorly and medially between the liver and IVC, reaching posterior to the duodenum and medial to the gallbladder. There is free intraperitoneal air and minimal free fluid in the pelvis and paracolic space.

Subhepatic and pelvic drains were removed due to minimal drainage, and a pigtail catheter, in the subcapsular space, was inserted under radiological guidance on the tenth postoperative day to drain the collection. The thoracic surgeon removed the thoracostomy drain on the 15th postoperative day, after the drainage stopped. There was no bile leakage, but sanguineous drainage of 600-800 mL per day continued from the pigtail catheter, with a drop in hemoglobin to 10 gm. CT scan with intravenous contrast was repeated on the 17th postoperative day, which showed a suspected pseudoaneurysm (Figures 4, 5).

**Figure 4:**
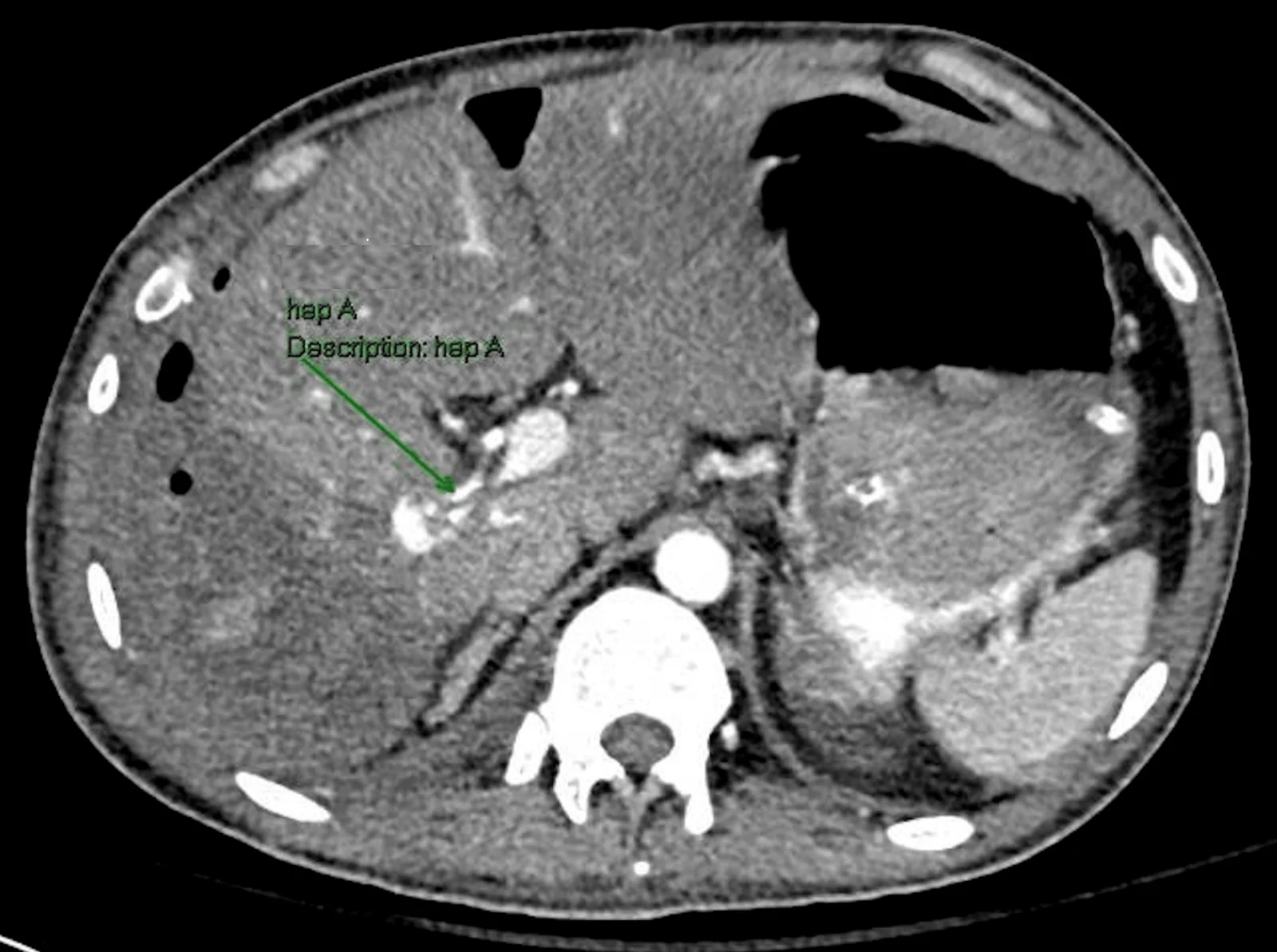
Contrast-enhanced CT liver Fusiform areas adjacent to the right portal vein, suspected pseudoaneurysm of the right hepatic artery, marked by an arrow.

**Figure 5:**
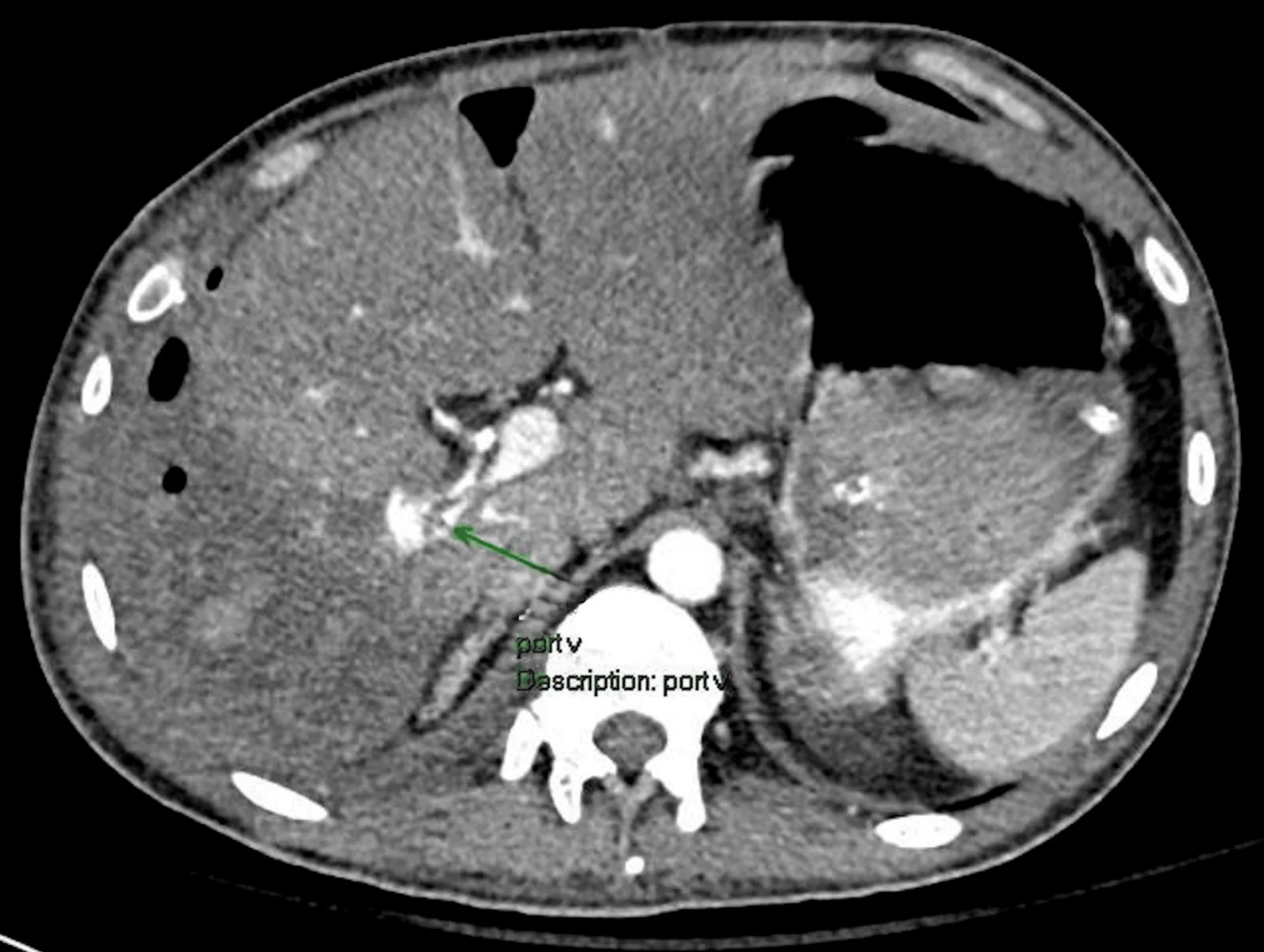
Contrast-enhanced CT liver Fusiform areas adjacent to the right portal vein, marked by an arrow.

Transarterial embolization was planned. The next day, under twilight sedation and aseptic precautions, a small incision was made in the right groin. Using the percutaneous Seldinger technique, a C2 5Fr catheter was guided through a vascular sheath, into the right femoral artery, right common iliac artery, aorta, celiac trunk, and then into the right hepatic artery over a 0.035-inch Terumo (Terumo Corporation, Tokyo, Japan) guidewire. A microcatheter 2.8Fr Excelsior-1018 (Stryker Neurovascular, Fremont, CA, USA), over a 0.014-inch Traxcess (Terumo Neuro, Aliso Viejo, CA, USA) micro-guidewire, was passed. Hepatic angiogram confirmed the findings on CT (Figure 6).

**Figure 6:**
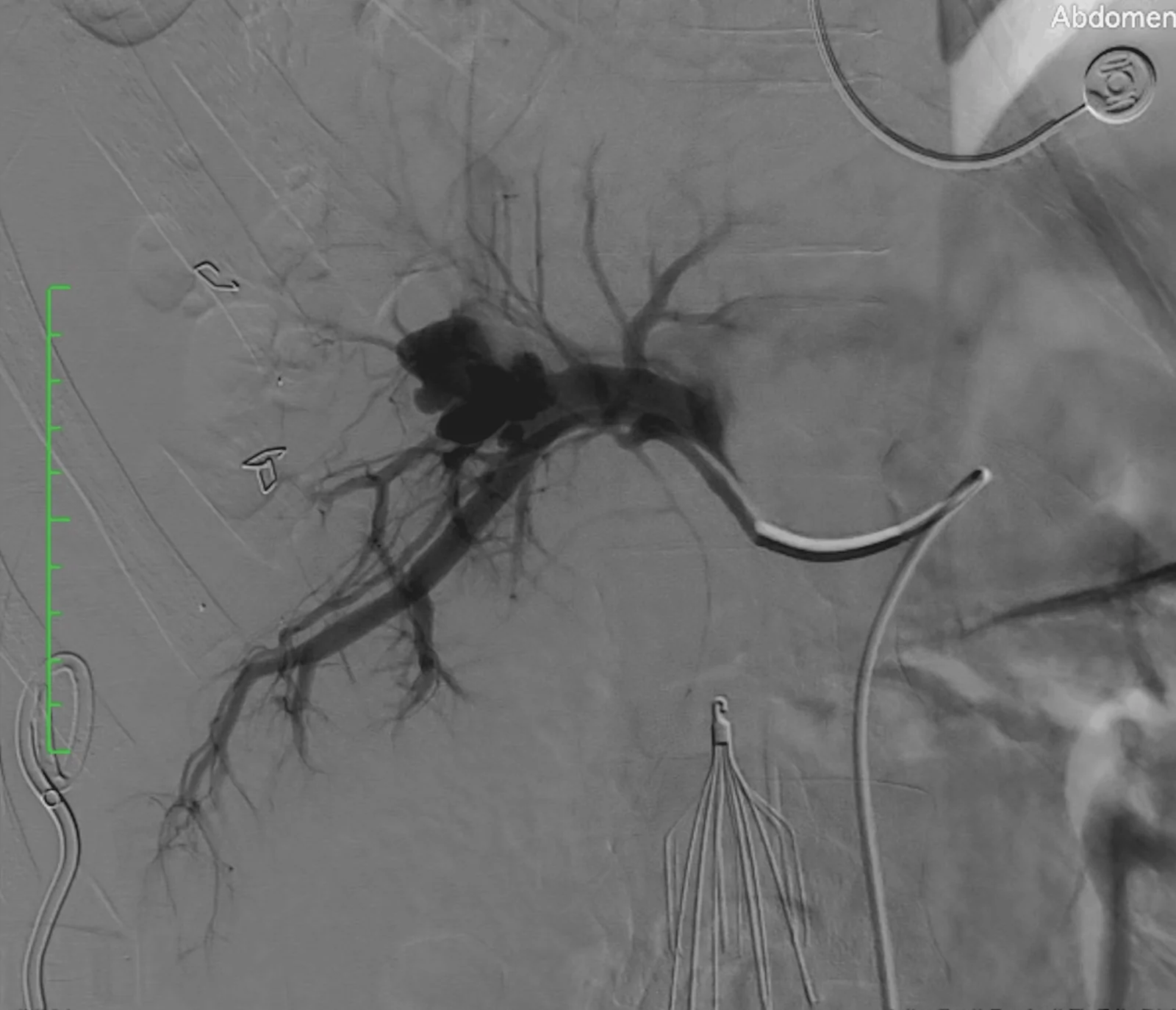
Hepatic angiogram Saccular pseudo-aneurysm arising from the posterior segmental branch of the right hepatic artery and fistula formation with the posterior branch of the right portal vein.

The radiologist started coiling with detachable micro coils 5 & 6 mm due to the small size of the vessels. Target coils were deployed, followed by pushable coils. Embolization was done with the sandwich technique, blocking both inflow and outflow vessels and filling the sac of the pseudoaneurysm with three coils (Figure 7).

**Figure 7:**
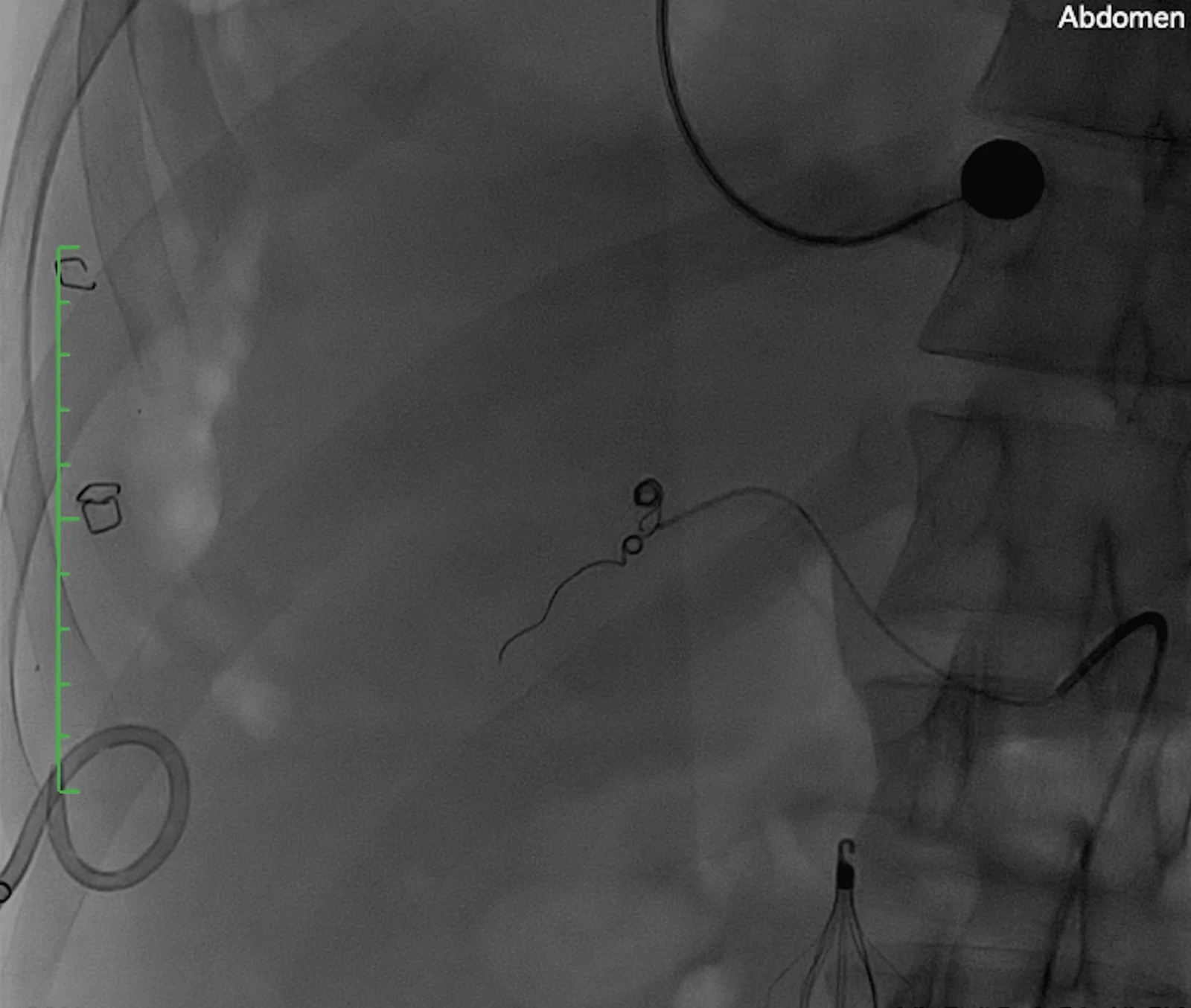
Transarterial embolization The coils are in place.

The patient tolerated the procedure well and exhibited a mild rise in liver enzymes, which settled in three days, with no audible murmur in the hepatic area. Enoxaparin 4000 IU subcutaneous was started. The patient had normal liver enzymes and stable hemoglobin levels on follow-up laboratory studies after four days. The patient was weaned off the mechanical ventilator and extubated. He was shifted to the surgical ward when he tolerated oral feeding. After two weeks post-procedure (embolization), an MRI abdomen was done showing no complication of embolization. Contrast-enhanced CT was repeated after five postoperative weeks (Figure 8).

**Figure 8:**
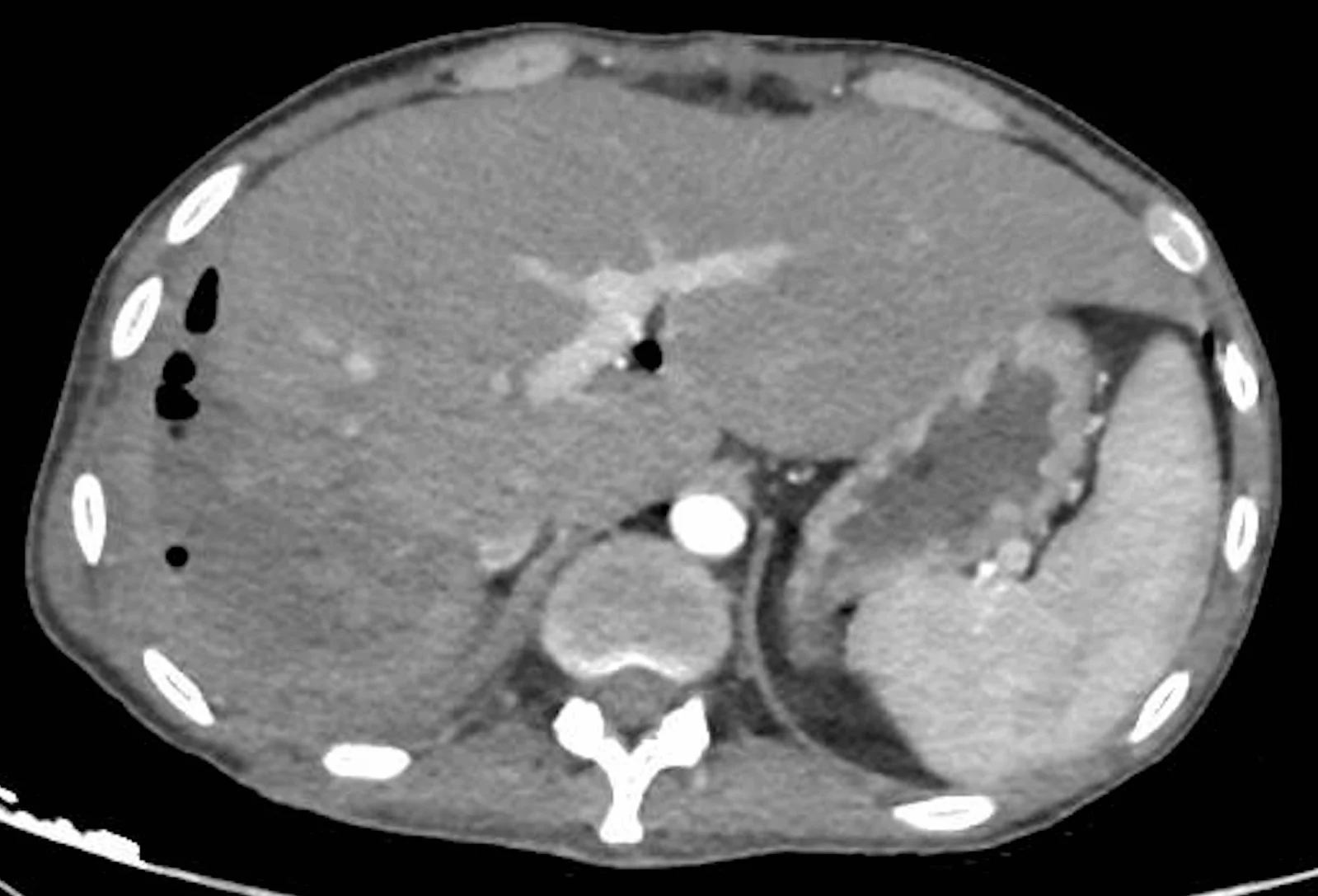
Contrast-enhanced CT liver The liver demonstrated normal homogeneous attenuation, without any evidence of perfusion abnormality or infarction. No evidence of contrast extravasation (active bleeding). Right hepatic subcapsular hematoma decreased in size.  Patent portal vein and its central branches as well as hepatic veins.

Subcapsular collection was later monitored by abdominal ultrasound. After drainage stopped, a pigtail catheter was removed at six weeks, and the patient was discharged home in a stable condition after an uncomplicated hospital course. He was readmitted after one month, and the IVC filter was removed.

## Discussion

A hepatic artery pseudoaneurysm with a fistula to the portal vein is a rare, high-flow vascular anomaly. The iatrogenic causes (endoscopic retrograde cholangiopancreatography, transhepatic biopsy, or surgery) are responsible for 50%, while 20% are due to trauma, and 20% are from rupture of a hepatic artery aneurysm into the portal vein [7]. IHPA may give no symptoms and be incidentally discovered on CT/angiography to diagnose other pathologies [8]. Sometimes they present as abdominal pain and complications of portal hypertension, months or years after liver trauma [9]. The diagnosis of hepatic pseudoaneurysm often relies on imaging, computed tomography, and digital subtraction angiography [10]. CT angiography outlines the vessels and associated complications. Our patient had a subcapsular hepatic collection with increasing volume of drainage from a pigtail catheter. The diagnosis was suspected on contrast CT and confirmed on angiography.

The pseudoaneurysms gradually enlarge, and post-traumatic IHPA bears a 44% risk of rupture leading to massive bleeding with high mortality. Prophylactic interventions by angioembolization are imperative to prevent rupture [11], as well as to treat ruptured pseudoaneurysms [12]. It is highly effective in controlling hemorrhage in stable patients with hepatic injuries and contrast extravasation, but carries a known risk of liver-related complications (LRCs) [13]. Depending upon the nature of the injury and technical factors, stent grafts, coils, microspheres, or absorbable gelatin sponge can be used to control the hemorrhage. Coil emboli slow blood flow through mechanical blockage and induce thrombosis in the proximal part of the vessels. The aim of embolization is selective closure of the fistula while preserving the adjacent normal vasculature, which directs the characterization of the collateral vessels that supply blood to the hepatic segments. We treated our patient with selective angioembolization with coils by the sandwich technique, which has a success rate of over 90%. The subcapsular collection responded well to endovascular treatment. The pseudoaneurysm was located in a tortuous, segmental branch of the right hepatic artery, and the highly flexible micro-catheters easily delivered the coils to the target. The stiff delivery systems of covered stents cannot navigate the small vessels and are reserved for larger proximal arteries such as the main hepatic artery. Moreover, the possibility of organ necrosis after coil embolization is lower compared with that after stent placement [14].

Noorbakhsh et al. retrospectively analyzed 127 patients with penetrating liver injuries (AAST - Grades IV-V) between January 2019 and October 2022 [9]. They identified acute hepatic arterio-portal fistula in five patients. Four patients presented with hemorrhagic shock and underwent emergency surgery. Postoperative angiography and angioembolization with coils were done in one patient, and with gelatin sponge particles in three patients. The fifth patient, after identification of the hepatic arterio-portal fistula, was sent directly for angiography and embolization with gelatin sponge. Follow-up CT showed resolution of the fistula in all five patients. Sgalambro et al. analyzed all patients from January 2010 to December 2019 in a single center who were treated in emergency by intra-arterial embolization for hepatic or renal hemorrhage [4]. Bleeding from one artery in 20 cases (66.7%), from two in four cases (13.3%), and multiple hemorrhagic sources in six cases (20%) were successfully embolized. Complete embolization was achieved with coils in 12 patients (40%), and with hemostatic sponges in 18 patients (60%). Second embolization was needed in one patient only. No major post-procedural complications were recorded. The mean procedure time was 65.1 minutes.

Han et al. reported a 29-year-old male with a stab wound to the abdomen who presented with a pseudoaneurysm of the hepatic artery and a fistula with the portal vein after five years [15]. The patient presented with symptoms of acute gastroenteritis with watery diarrhea and later with hematemesis and hematochezia. They found early opacity of the portal vein on contrast CT, and hepatic arteriography confirmed a high-flow connection between the left hepatic artery and the left portal vein branch. They successfully embolized the fistula with five fibered coils. During the six-month follow-up period, the patient remained asymptomatic. Hassine et al. reported a 25-year-old patient who sustained blunt abdominal trauma after a road traffic accident with liver contusion and later developed Quincke's triad, suggesting pseudoaneurysm rupture [1]. CT scan demonstrated significant contusion in segment IV of the liver. Intravenous contrast in the arterial phase exhibited an aneurysmal sac arising from the right hepatic artery. Under fluoroscopic control, percutaneous selective embolization of an aneurysmal sac was done, while patency of the main hepatic artery and its branches was maintained.

Complications of angioembolization are mentioned in the literature, including portal venous thrombosis or post-embolization syndrome of abdominal pain, fever, and ileus [16], bile leaks, hepatic/perihepatic abscesses, and necrosis due to liver ischemia. The frequency of LRCs after angioembolization is close to 30% [2]. Serious complications are usually recorded after embolization of the main hepatic artery, while embolization of segmental hepatic arteries is associated with fewer complications. Nguyen et al. compared embolization of the main hepatic artery (MHAA) with segmental arteries (SHAA) in 55 patients enrolled from 23 trauma centers across the US and Canada [17]. They concluded that selective hepatic artery embolization is preferable for liver injury with contrast extravasation, and the location of embolization, proximal versus selective, has a significant part in the development of complications, independent of the initial severity of the hepatic insult. Our patient had only a transient rise in liver enzymes, which settled in three days. Follow-up CT scans and ultrasound exhibited no bile leakage or liver necrosis. Our patient was highly satisfied of success of treatment without any serious complications.

## Conclusions

Post-traumatic hepatic artery pseudoaneurysm with fistula formation with portal vein is a rare complication of hepatic trauma. They need immediate attention. Selective angio-embolization may play a key role in controlling the complications related to fistula.
